# Investigation of the Effect of Imatinib and Hydroxyurea Combination Therapy on Hematological Parameters and Gene Expression in Chronic Myeloid Leukemia (CML) Patients

**DOI:** 10.3390/jcm11174954

**Published:** 2022-08-24

**Authors:** Esraa K. Al-Amleh, Ola M. Al-Sanabra, Khalid M. Alqaisi, Moath Alqaraleh, Jumana Al-Nahal, Lama Hamadneh, Mohammed Imad Malki, Jehad F. Alhmoud

**Affiliations:** 1 Department of Medical Laboratory Sciences, Faculty of Allied Medical Sciences, Al-Ahliyya Amman University, Amman 19328, Jordan; 2 Pharmacological and Diagnostic Research Center (PDRC), Al-Ahliyya Amman University, Amman 19328, Jordan; 3 Department of Medical Laboratory Sciences, Faculty of Science, Al-Balqa Applied University, Al-Salt 19117, Jordan; 4 Department of Biopharmaceutics and Clinical Pharmacy, Faculty of Pharmacy, Al-Ahliyya Amman University, Amman 19328, Jordan; 5 Faculty of Pharmacy, Al-Zaytoonah University of Jordan, Amman 11733, Jordan; 6 College of Medicine, QU Health, Qatar University, Doha P.O. Box 2713, Qatar

**Keywords:** BCL2, CIP2A, CML, gene expression, hydroxyurea, imatinib, leukemia, PP2A, WT1

## Abstract

(1) Background: Chronic myeloid leukemia is defined as the neoplastic development of mostly myeloid cells in the bone marrow. Several treatments, including chemotherapy, radiation, hormone treatment, and immunological therapy, can be used to control this condition. The therapeutic impact on leukemic individuals varies, and the response to therapy varies between patients due to disease heterogeneity. The primary goal of this study is to compare the effects of single and Imatinib (IM) and Hydroxyurea (HU) combined treatment on hematological parameters and gene expression in CML patients. (2) Methods: This study was conducted on 51 patients, with chronic myeloid leukemia, who were admitted to Al-Basher hospital in Amman, Jordan, for follow-up. Their hematological parameters were checked and gene expression was measured for (BCL2, PP2A, CIP2A, and WT1). (3) Results: The BCL2 gene was found to be less expressed in both IM and (HU + IM) treatments as compared to the HU group alone, while PP2A gene expression was raised. Such a thing indicates that the outcome of the combined therapy method is not ideal, since PP2A activation causes CML cells to move toward the blast crisis stage. Furthermore, CIP2A gene expression revealed that IM and (HU + IM) had the same therapeutic effect and were more successful in CML patients than HU alone. With regards to the treatment effect on hematological parameters, notably in CML patients in later stages, the combination therapy (HU + IM) raised lymphocyte count, indicating a greater response to the treatment. When compared to single medicines, the combination treatment reduced the proportion of neutrophils to normal reference ranges. Platelet counts, on the other hand, dramatically decreased in both IM and (HU + IM). (4) Conclusion: Because the studied genes (BCL2, PP2A, CIP2A, and WT1) are participating in cell proliferation and death, the findings show that the examined genes are significant to understand the efficacy of various therapies. Furthermore, it was found that there was a clear effect of the clinic-based strategic treatment on hematological indicators such as WBCs, lymphocytes, neutrophils, and platelet counts.

## 1. Introduction 

### 1.1. Cancer and Leukemia

Cancer is one of the major reasons of death, as, among every eight people worldwide, one dies due to one type of cancer [1]. These different types are characterized by a relatively uncontrolled cell proliferation that can invade body tissues, replace normal cells, and metastasize to distant organs [2]. Cancer occurs when aberrant cells divide uncontrollably and diffuse to other tissues in the body. The disease appears when genetic mutations disrupt the order of the cell proliferation process. Based on histological features, the disease is divided into five major types: carcinoma, sarcoma, myeloma, leukemia, and lymphoma [3].

Leukemia is a malignant leukocyte clonal proliferation that directly affects cell maturation and production in the bone marrow. This disease is generated from lymphoid or myeloid lineages and may be acute (precursor cell) or chronic (mature cell) subtypes. The onset of acute leukemia is quick, with fast progression, and a lethal end in a few weeks, compared with chronic leukemia [4]. This disease is also characterized by a frequent increase in the count of white blood cells (WBC), and the rate of proliferation in both mature and maturing cells of a particular lineage which leads to cell accumulation. Leukemia cells gradually replace most normal hematopoietic ones in the bone marrow, compromising normal bone marrow function. At the time of diagnosis, the symptoms of chronic leukemia are often obscure and varied [5].

### 1.2. Chronic Myeloid Leukemia (CML)

CML is a hematopoietic cancerous stem cell driven by the BCR-ABL fusion protein. The disease appears at an indolent chronic phase that can last for 3 to 5 years [6]. CML is known as chronic granulocytic leukemia (CGL), chronic myeloid leukemia, and chronic myelocytic leukemia. It is primarily characterized by the neoplastic growth of myeloid cells in the bone marrow. Erythrocytic and megakaryocytic lineages can expand, and the extramedullary granulocytic proliferation is localized in the spleen and liver, and that reflects disease progression [7]. CML is divided into chronic, rapid, and blast crises. Traditional chemotherapy is used to restore and maintain the normal division rate for months and up to several years. Comparatively, 70–90% of the treated patients in the chronic phase, with tyrosine kinase inhibitors (imatinib mesylate), experienced a 5-year progression-free survival [7].

### 1.3. Treatment of CML

Hydroxyurea (HU) is the first-line preferred option to be used for a patient with suspected CML and an increased WBC count (e.g., >80–100 × 10^9^/L), to decrease the WBC count to normal levels [8]. The treatment protocol suggests giving the patients HU until confirming the presence or absence of the Philadelphia chromosome. In cases where the WBC is exceedingly high, allopurinol is taken to reduce the risk of some problems generated by tumor lysis. After the diagnosis is confirmed, the patient directly receives Imatinib, a tyrosine kinase inhibitor (TKI). Generally, it is not required to maintain the count of WBC at normal levels before using TKIs, and HU should be stopped once the treatment with TKI starts [8,9].

Imatinib (IM) was developed to target BCR-ABL1, which is constitutively active in chronic myeloid leukemia (CML) [9]. It is authorized by the FDA for the treatment of chronic myelogenous leukemia and gastrointestinal stromal tumors. In addition, this treatment has been studied and applied to a variety of malignant cancers [10]. Imatinib Mesylate has the chemical formula 4-4 ((4-methyl-1-piperazinyl) methyl)-N-(4-methyl-3-((4-(3-pyridinyl)-2-pyrimidinyl) amino) phenyl) -benzamide mono methane sulfonate (IMT). Its chemical formula is C29H31N7O.CH4O3S and the molecular weight is about 589.71 g mol^−1^ [11].

HU, also known as hydroxycarbamide, is a non-alkylating antineoplastic and antiviral drug that has been used to treat a broad range of health problems interrelated with hematology, oncology, contagious diseases, and dermatology. A study discovered that it might be utilized to treat a variety of solid tumors and myeloproliferative diseases [12]. HU is a white or almost white powder that is crystalline and hygroscopic. The chemical formula of this medication is CH4N2O2 and has a molecular weight of 76,055 g mol^−1^ [13].

Although newer and more efficient treatments have replaced HU in certain cases, it is still used in clinics as a proven, dependable, and well-tolerable small-molecule medication for a variety of neoplastic and non-neoplastic disorders [14]. It is being used to treat sickle cell anemia and chronic myeloproliferative diseases, and is classified by the World Health Organization as an essential therapy [15].

### 1.4. Genes Involved in CML

PP2A is an essential protein phosphatase that regulates most cellular processes by forming holoenzyme that control cell proliferation, survival, and differentiation. It provides more than 90% of the cell’s phosphatase activity along with PP1. PP2A is routinely inactivated in both AML and CML. Numerous studies currently focus on PP2A inactivation as a prerequisite for human cell transformation. 

CIP2A protein, also known as a carcinogenic inhibitor of PP2A, is a protein that is found mainly in humans. In human cancers, CIP2A reduces PP2A’s tumor suppressor action. In particular, this has been shown that blocks PP2A activity towards the oncogenic transcription factor c-Myc, leading to prevent the proteolytic breakdown of c-Myc. Furthermore, CIP2A is necessary for malignant cellular proliferation and tumor development in vivo. Overexpression of CIP2A enhances Ras-elicited cell proliferation and changes immortalized human cells, which are consistent with CIP2A’s oncogenic activity (HEK-TERVs) [16]. 

B cell lymphoma 2 (BCL2) is a key participant in the eukaryotic cell’s genetic program that promotes survival by suppressing cell death. BCL2 protein overexpression has been observed in a variety of human malignancies, including leukemia, lymphomas, and carcinomas. The mechanism of BCL2 activation in follicular lymphomas and a subset of BCLs diffuse were discovered to be the translocation t(14,18)(q32;q21), which results in uncontrolled gene expression [17].

Wilms’ tumor gene (WT1) encodes a transcription factor that is essential for cell proliferation and differentiation. The WT1 gene is strongly expressed in leukemia and other forms of solid tumors. This gene is a useful tumor marker for detecting leukemia’s minimum residual illness; it was initially thought to be a tumor suppressor gene, but it was found to be an oncogene [18].

The major goal of this study is to evaluate the effect of single and combined chemotherapy of IM and HU on hematological parameters and gene expression in CML patients that distinguish the pathophysiology of this disease.

Our findings might give more insights into the effectiveness of treatment protocols followed in clinics, and whether these therapeutics are potent in controlling disease progression.

## 2. Materials and Methods

### 2.1. Ethical Approval

This study was performed in line with the principles of the Declaration of Helsinki. Approval was granted by the scientific research committee of Al-Ahliyya Amman University (IRB: AAU/3/8/2021–2022).

### 2.2. Consent to Participate

Informed consent was obtained from all 51 participants included in the study.

### 2.3. Patient Cohort Criteria

A total of 51 patients, males and females, who were diagnosed with CML, participated in this study. The inclusion criteria of the samples were as follows: patients diagnosed with CML according to the World Health Organization (WHO) 2008 classification: ages about 20 years old and above; patients after single-agent induction of chemotherapy (HU); and those who received both treatment agents (IM and HU) chemotherapy. All patients were treated with standard induction chemotherapy. The CML newly diagnosed patients (with no treatment), together with those who were previously diagnosed, were both treated with a short dose of HU and a long dose of IM. 

The exclusion criteria for this studied cohort included the following: any missing clinical data related to the type of treatment other than IM or HU or a combination of IM and HU; patients less than 20 years old; and patients who were hospitalized. 

### 2.4. Patients’ Collection Sample and Ethical Consideration

**Figure 1:**
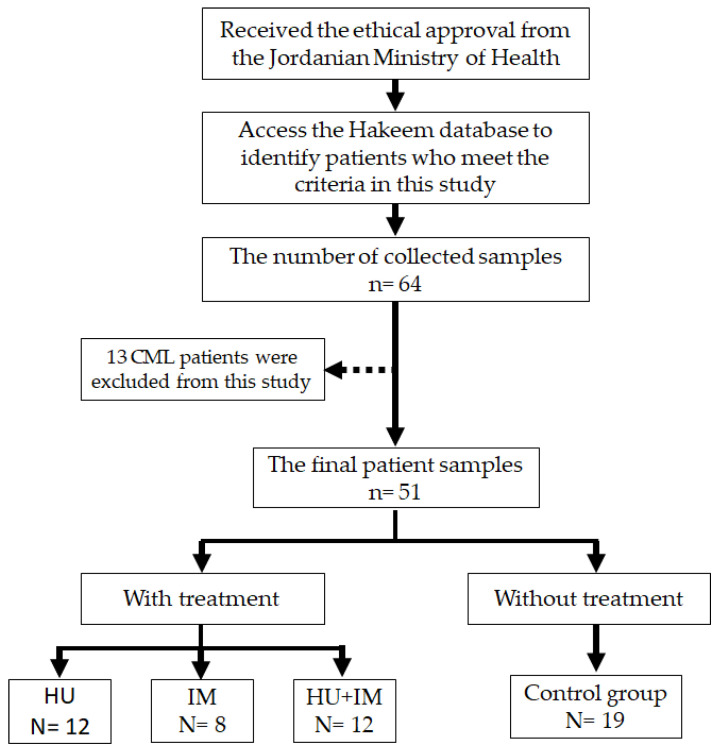
The flow chart illustrates our research strategy for obtaining the final sample.

Venous blood samples of patient cohorts who meet the inclusion criteria were collected throughout August-November 2021 (Figure 1). Those patients were admitted to Al-Basher hospital in Amman, Jordan, for follow-up. The blood samples were immediately processed for both hematological and gene expression (BCL2, PP2A, CIP2A, and WT1). The study comprised blood samples of 51 patients diagnosed with CML and those who had already received treatment. The population size was chosen based on Statistics from the Jordanian Ministry of Health, regarding the prevalence of CML disease in Jordan. 

### 2.5. Gene Expression

#### 2.5.1. Extraction of Ribonucleic Acid (RNA) and Synthesis of Complementary Deoxyribonucleic Acid (cDNA) 

Zymo Research’s Direct-zol RNA miniprep kit was used to extract the RNA, Lot No. ZRC206161/USA). cDNA was synthesized from 1 ng/mL of total RNA using a primeScript RT master mix (Takara- Lot No. AK22353A/San Jose, CA, USA), according to the manufacturer’s protocol.

#### 2.5.2. Primer Design

In all genes, the sequence of their primers was designed using primer blast, except for CIP2A, which was taken from a scientifically published paper and used as a reference [19], as illustrated in Table 1. The designed primers were ordered by a commercial provider (IDT, Coralville, IA, USA). A strict set of criteria was employed for primer design to achieve optimum specificity and efficiency during PCR amplification.

After selecting the optimal annealing temperature, based on the information mentioned above, optimization steps were performed.

#### 2.5.3. Gene Expression Analysis

According to the manufacturer’s instructions, quantitative real-time PCR (qPCR) was used to determine gene expression in leukemic patient cells using (SsoAdvanced Universal SYBR Green Supermix/ BIO-RAD, Hercules, CA, USA) (Lot No 1725274). As a result, the expression of these genes assessed by real-time quantitative PCR (qPCR) was standardized to that of β-actin. The CFX96 real-time PCR detection device was used to perform RT qPCR in 96-well plates (Bio-Rad, Hercules, CA, USA).

For each gene, two independent technical replicates were employed. The identical RNA preparation was used to amplify all cDNA samples in triplicate, and the mean value was used (i.e., two technical replicates). To prevent differences between runs, all samples for each analyzed gene were performed on the same plate. The CFX Manage version 1.6 software (Bio-Rad, Hercules, CA, USA) generated the baseline and Ct values automatically, using the default settings.

The thermal profile and details of RT-PCR programs for the four genes BCL2, PP2A, CIP2A, and WT1 were optimized based on the literature and manufacturer recommendations. 

### 2.6. Statistical Analysis

This study was conducted on 51 chronic myeloid leukemia patients: 19 patients without treatment and 32 patients with different treatment protocols. For data analysis, GraphPad Prism version 5 statistical software was used. 

The variance for each studied gene was calculated besides the standard deviation and the standard deviation of the reference gene, which was normalized to the exponential arithmetic mean of the ct value, in comparison with the reference genes. The patients were divided into four groups. The first group included patients without treatment (control); the second included patients with HU; the third was that of patients who were treated with IM therapy; and the fourth included patients under combination therapy (HU + IM). First, the Δct of the target gene, and that of the housekeeping one, were calculated, then the ΔΔct was calculated in addition. The two-way paired *t*-test was performed and used to calculate and consider the null hypothesis to determine whether there is a relationship between the genes or not.

A paired *t*-test was applied to assess the relationship between different hematological parameters in all patient categories. These parameters include: lymphocytes, neutrophils, basophils, platelets, and RDW-SD. The results were taken into consideration if the *p*-value was less than or equal to 0.05.

Correlation analysis was performed to detect the significant relationship between different gene expressions in the same patient group (UT, HU, IM, HU + IM).

## 3. Results 

### 3.1. Patient Cohort Demographic Data

This study was conducted on 51 chronic myeloid leukemia patients, 15 males and 36 females. The patients’ ages ranged between 21 to 70 years. All of them met the inclusion criteria included in this study. The study participants consist of four groups: CML without treatment (control group) (n = 19, M = 4, F =15); CML patients with HU treatment (n = 12, M = 1, F = 11); CML patients with IM treatment (n = 8, M = 6, F = 2); and CML patients with combination therapy (HU + IM) (n = 12, M = 4, F = 8). HU + IM; HU, and IM. None of the patients have a family history of CML. The error bars indicate the standard error, n = 51.

### 3.2. Gene Expression

In this section, we focused on comparing the relative expression fold for each studied gene among different patient categories.

#### 3.2.1. BCL2 Expression

**Figure 2:**
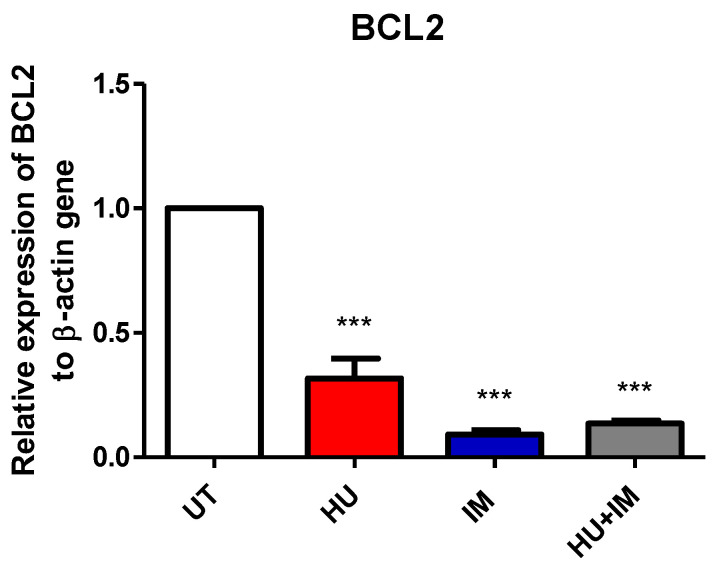
The relative expression of BCL2 gene to β-actin gene. Two-tailed paired *t*-test revealed a significant statistical difference between UT vs. HU, UT vs. IM and UT vs. HU + IM (*** *p* < 0.005). (mean ± SEM; n = 2).

The relative expression fold for the BCL2 gene was measured using RT-PCR. Both primer and housekeeping genes were run twice and the average was calculated, then the average of ∆∆Ct for each CML patient category (UT, HU, IM, HU + IM) was measured (Figure 2). All treated groups were statistically compared with the UT CML patients to observe the effect of treatment on gene expression. The results showed that the Bcl2 gene had low expression in both IM and HU + IM treatments, compared with other groups. This indicated that the apoptotic effect of the combination is the same as the treatment with a single agent (IM), which revealed that using the combination therapy has no significant effect compared with IM. Therefore, using IM alone is enough to have an impact on cancer cells.

#### 3.2.2. PP2A Expression 

**Figure 3:**
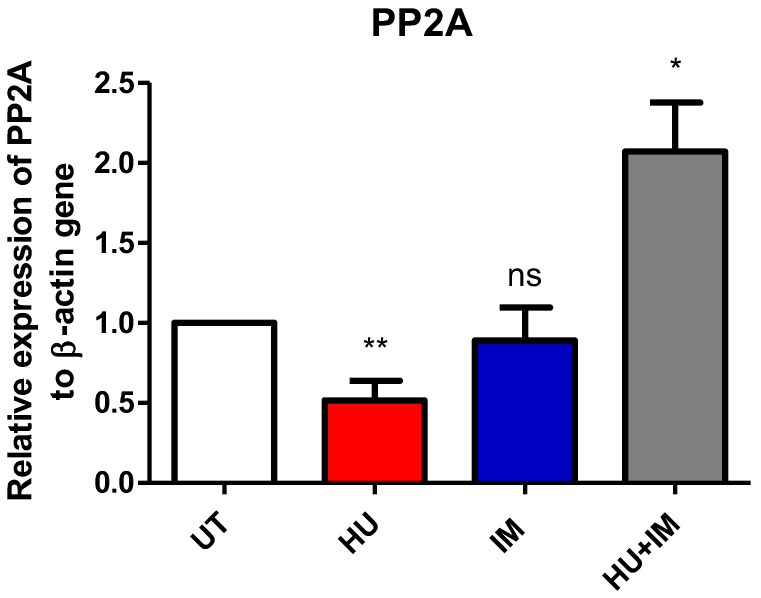
Relative expression of PP2A to β-actin gene. Two-tailed paired *t*-test showed a significant statistical difference between UT vs. HU (** *p* < 0.01), and UT vs. HU + IM (* *p* < 0.05). (mean ± SEM; n = 2).

The relative expression fold for the PP2A gene was measured using RT-PCR. Both primer and housekeeping genes were run twice and the average was calculated, then the average of ∆∆Ct for each CML patient category (UT, HU, IM, HU + IM) was also measured (Figure 3). All treated groups were statistically compared with the UT CML patients to observe the effect of treatment on gene expression.

This figure shows that the PP2A gene has a significant increase in the relative expression fold in the combination group, compared with other groups. This indicates that the outcome of combination therapy is not preferable in the treatment strategy, as the activation of Pp2a leads to an increase in the progression of CML cells into the blast crisis stage. Therefore, using HU as a single agent has a more potent effect on inhibiting the proliferation of cancerous cells. The findings confirmed that CML patients under HU have a lower relative expression fold of Pp2a, which improved the disease response to treatment.

#### 3.2.3. CIP2A Expression

**Figure 4:**
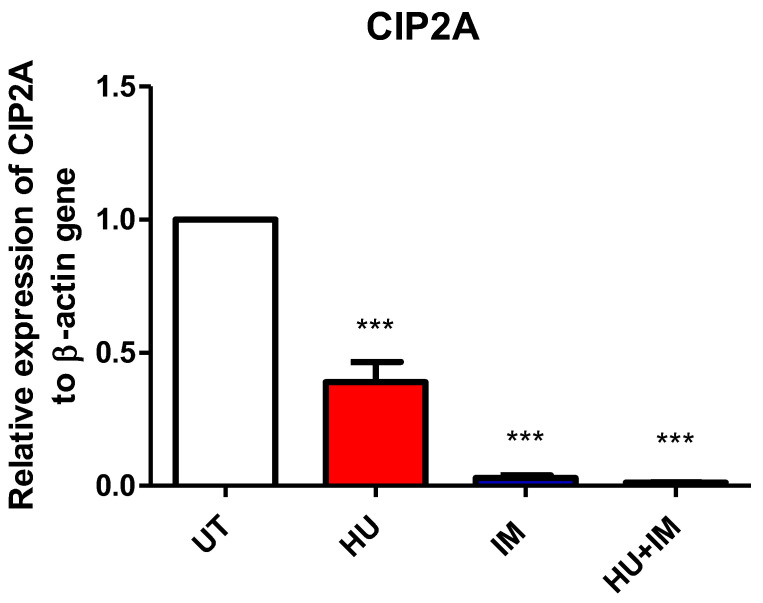
Relative expression of CIP2A to β-actin gene. Two-tailed paired *t*-test showed a significant statistical difference between UT vs. HU, UT vs. IM, and UT vs. HU + IM (*** *p* < 0.005). (mean ± SEM; n = 2).

The relative expression fold for the CIP2A gene was measured using RT-PCR. Both primer and housekeeping genes were run twice and the average was calculated, then the average of ∆∆Ct for each CML patient category (UT, HU, IM, HU + IM) was measured as well (Figure 4). All treated groups were statistically compared with the UT CML patients to observe the effect of treatment on gene expression. 

The results indicated that both IM and HU + IM have the same treatment effect and are more potent in CML patients, compared with HU alone. These findings suggest that using IM or HU + IM is recommended to improve the patient outcome as they inhibit CIP2A gene expression involved, in regulating cell proliferation and increasing cell death.

#### 3.2.4. WT1 Expression

**Figure 5:**
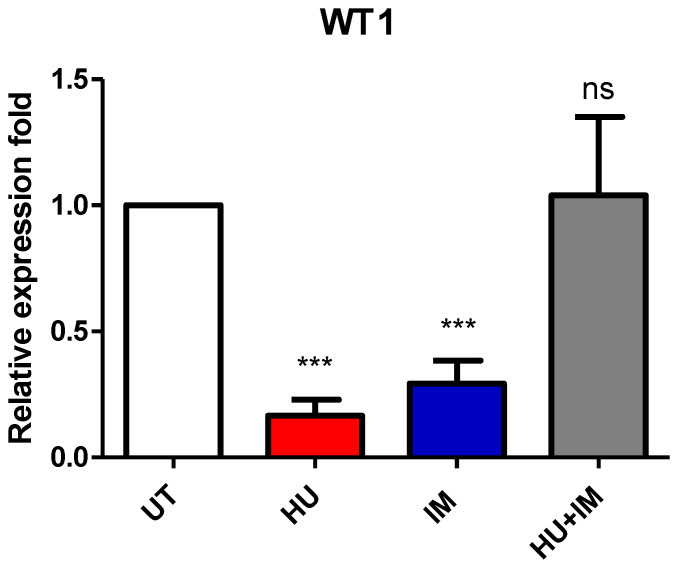
Relative expression of WT1 to β-actin gene. Two-tailed paired *t*-test showed a significant statistical difference between UT vs. HU and UT vs. IM (*** *p* < 0.005). (mean ± SEM; n = 2).

The relative expression fold for the WT1 gene was measured using RT-PCR. Both primer and housekeeping genes were run twice and the average was calculated, then the average of ∆∆Ct for each CML patient category (UT, HU, IM, HU + IM) was measured as well (Figure 5). All treated groups were statistically compared with the UT CML patients to observe the effect of treatment on gene expression. 

As the relative expression fold of WT1 can be considered a marker for cancer progression, decreasing its expression may, therefore, play a role in stabilizing the disease status in CML cases. Based on the above findings, utilizing different single-agent protocols (HU or IM) seems to be much more beneficial than combination therapy (HU + IM). The results of WT1 in the combination therapy are similar to those in the patients without treatment. This suggests finding out other combinations to reduce the WT1 expression, particularly in CML patients in later stages.

### 3.3. Hematological Parameters for Study Patients

**Figure 6:**
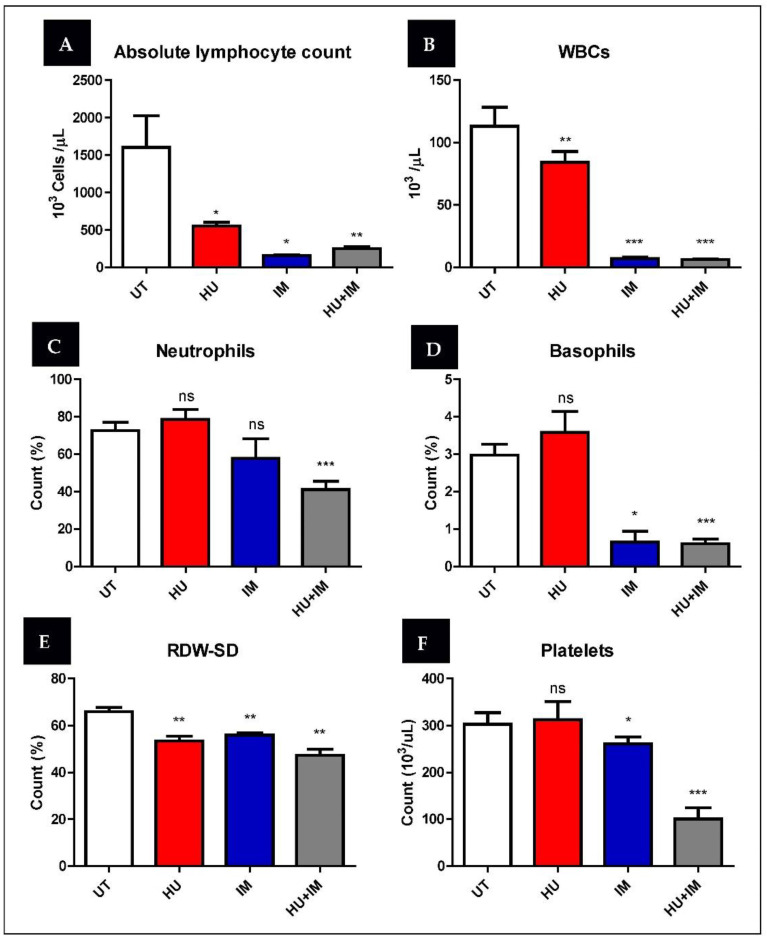
Hematological parameter changes due to IM, HU, IM, and HU treatment of CML patients. (**A**) absolute lymphocyte count; (**B**) WBC’s; (**C**) neutrophils; (**D**) basophils; (**E**) RDW-SD; (**F**) platelets. Two-tailed paired *t*-test was performed and the statistical difference between the untreated and treated patients is shown in the figures (* *p* < 0.05, ** *p* < 0.01, *** *p* < 0.005).

Several hematological parameters are shown below (Figure 6). They were selected based on the significant change, whether an increase or decrease, in their values compared with the control (UT) group after receiving treatments (HU, IM, HU + IM). The average was calculated in all patient categories (UT, HU, IM, HU + IM) for each hematological parameter. 

(A) The number of absolute lymphocytes was decreased with the combination therapy (HU+IM), indicating a better response to the treatment compared with other single-agent therapy. (B) The WBC count was decreased significantly in the combination and IM therapy, and the effect of both treatments on the WBC count was more than the treatment with HU alone. (C) The combination therapy decreased the percentage of neutrophils to the normal reference intervals compared with single agents. (D) The percentage of basophils was maintained in the normal range in both (IM, HU + IM). (E) RDW-CV values indicated normal cell population size after treatment, especially after treatment with combination therapy. (F) Platelet numbers were reduced, particularly in both (IM, HU + IM). 

### 3.4. Correlation Analysis of Gene Expression

A correlation analysis was performed to understand the pattern of the relationship between expressions of the different genes discussed in this study (Table 2). Only the CML patients who received treatment were included in this section. 

The correlation analysis revealed that there is an inverse relationship between all treatments of PP2A vs. CIP2A, BCL2 vs. PP2A, and WT1 vs. PP2A. There is also a positive relationship between CML patients who received IM treatment in BCL2 vs. CIP2A, WT1 vs. CIP2A, and WT1 vs. BCL2.

## 4. Discussion

Several types of research were published over the past few years that focused on understanding the potency of cancer therapies through observation of the expression of the targeted genes [20]. The results could give more insight into predicting disease prognosis and treatment response. The overexpression, or low expression, of some key genes is involved in inducing the proliferation of cancerous cells, in addition to preventing the rate of apoptosis [21].

Recently, pharmacological development and therapeutic methods have improved at an accelerating and remarkable rate. The factor that plays the most important role in this aspect is knowledge increase and the understanding of the pattern in which cancer cells divide and spread in the body [22]. This helps to produce more different types of drugs that are more targeted to get the maximum possible benefit, and minimize the cytotoxic effects after receiving treatment. 

In this study, two types of therapies were selected, based on the treatment strategy that is used in Jordanian hospitals for patients with Chronic Myelogenous Leukemia (CML). The mechanism of action of HU is that it is an efficient ribonucleotide reductase (RNR) inhibitor that diminishes intracellular deoxynucleotide triphosphate pools and effects as an S-phase-specific agent with DNA synthesis suppression, which ultimately leads to cell cytotoxicity. The main reason for using this agent in leukemia patients is to reduce or prevent the growth of tumor cells in the body [23]. IM is used in a clinical setting to prevent a BCR-ABL protein from exerting its role in the oncogenic pathway in CML. IM directly inhibits the substrate such as; GRB2 (Growth Factor Receptor Bound Protein 2) from entering the tyrosine kinase site, leading to the prevention of tumor cell proliferation [24].

Various gene expression techniques have been established, including: Reporter genes; Northern blotting; RNA Seq; DNA microarray; and reverse transcription-polymerase chain reaction (RT-PCR). The latter was conducted in this study, as this method is simple, easy to use, rapid, and cost-effective, and a smaller number of samples can be used with enormous specificity and sensitivity [7]. 

The BCL-2 relative expression fold has clearly shown that using a single agent of IM or combination therapy decreased the expression, indicating that both treatment strategies have raised the apoptotic effect in tumor cells. This finding agrees with the previous study which reported that using combination therapy, such as ABT-199 and IM that targets both BCL-2 and BCR-ABL tyrosine kinase, has a significant impact on regulating and improving CML patient stability in chronic phase and blast crisis phases [25].

The combination therapy (HU + IM) has an adverse effect on the CML patient in the blast crisis phase, as the relative expression fold of PP2A was high in this category of patients. Thus, using single-agent HU or IM is more effective, with fewer side effects, particularly in CML patients at late stages. In the literature, a study was conducted on leukemic stem cells which showed that inhibiting both PP2A and BCR-ABL (IM) may be a viable therapeutic approach for targeting a drug-insensitive for these cells, that leads to preserving MRD in patients [26].

Since CIP2A can be used as a marker for the pathogenesis of chronic myelocytic leukemia, our results showed that there was an impact of treatment on the relative expression fold for the CIP2A gene. The results also demonstrated that receiving IM or combination therapy in CML patients has a positive effect on inducing cell death and on regulating cell proliferation, which was observed from the down-expression of this gene. In addition, CIP2A indicates that it may reveal therapy resistance of tyrosine kinase inhibitors (IM). Therefore, the reduction in the expression of this gene gives a good impression of the treatment response [27].

The WT1 gene is usually used for monitoring the progression of BCR-ABL positive chronic myeloid leukemia. Our results showed that the patient who received combination therapy (HU + IM) had a bad prognosis, compared with the patient who had never received any treatment (UT). Based on these findings, it is highly recommended to alter the treatment protocol for the patient group (HU + IM) and to use a single agent (HU or IM) instead of the combination therapy (HU + IM). El-Menoufy, M. A. and Ahmed, M. A. revealed that repeated evaluation of WT1 transcript levels in CML patients might be a valuable marker for predicting early and abrupt disease progression [28].

On the other hand, hematological parameters were affected to varying degrees (increasing or decreasing) after receiving treatment. Total white blood cells were maintained in the normal range in both (IM, HU + IM). After receiving a single agent of IM or combination therapy (HU + IM), the total number of white blood cell counts has reduced and maintained in the normal range. This correlates with a published research article that reported that patients with low WBC and/or early MRD response may be treated with autologous SCT or long-term TKI therapy [29].

The patients who received (HU + IM) had a significant induce in absolute lymphocyte count. This result demonstrated that following the administration of a therapy containing IM, the number of B lymphocytes is expected to be activated and this could be considered as a marker of CML patients for treatment response, specifically if combined with a cytogenetic response. This conclusion completely matches the previous findings, the bone marrow CD20+ lymphocytes were increased after receiving IM therapy, due to the long-term effect of inhibiting the BCR-ABL kinase. The number of lymphocytes in the patients who received HU was reduced, which corresponds to its mechanism of action [30].

Administration of IM or (HU + IM), induced the reduction of both neutrophil and basophil counts and almost maintained the normal reference intervals. After receiving IM, the basophil count decreased to below 3%. A published study on a 57-year-old woman with chronic myeloid leukemia who had significant severe basophilia, revealed that after a few days of stopping IM, a remarkable decrease in basophils occurred; such as thing may result in severe cutaneous responses [30]. Furthermore, a research study conducted on a 42-year-old woman, diagnosed with chronic-phase CML Philadelphia, confirmed that receiving seven full months of IM dose leads to a decreased number of neutrophils to normal values. Patients who experienced myelosuppression required a dosage reduction, as they have a lower likelihood of responding to IM [31].

The number of platelets was markedly decreased especially after the administration of combination therapy (HU + IM). This finding copes with the mechanism of the treatment strategy through targeting and reducing myeloid cell count and affecting their function. Decreasing platelet count might lead to several complications including hemorrhage, so using a single agent HU or IM seems to be safer than the combination therapy. Akay, O. M. et al. mentioned that a high rate of chronic myeloid leukemia patients suffer from different types of platelet dysfunction that are not associated with the use of imatinib mesylate. IM also plays a role in causing a significant decline in ristocetin-induced platelet aggregation [32].

The red blood cell distribution width (RDW-CV) was elevated in the control patient group, as an increase in the blast cells in the bone marrow directly affects the production of RBCs, causing anemia. After the WBC count has been maintained, the size of the RBC population was induced to the normal range to compensate for the lost cells. Researchers have shown that RDW has a critical role in the health status of CML-CP patients for predicting treatment responses and outcomes and could assist in treatment planning. As such, patients with low RDW have a much better treatment response and a 5-year event-free survival rate compared with patients with high RDW levels [33].

A correlation analysis has been performed (see Table 2) to measure how closely two variables are connected. A correlational investigation can provide three outcomes: a positive correlation, a negative correlation, or no correlation. A positive correlation is a two-variable association in which both variables move in the same direction. As a result, when one variable rises, the other increases, or when one variable reduces the other declines. A negative correlation is a link between two variables in which a rise in one causes a decrease in the other. When there is no link between two variables, the correlation is 0, which means that there is no correlation detected.

Our correlation analysis results revealed that all patients who received IM had a positive correlation between BCL-2 vs. CIP2A, WT1 vs. CIP2A, and WT1 vs. BCL-2. This indicates that the reduction of their relative expression folds has a positive impact on stimulating growth arrest and apoptosis in malignant cells. Whereas all the previously mentioned genes negatively correlated to the relative expression folds of PP2A expression. These findings cope with the literature as activating PP2A leads to dephosphorylate BCL2 and/or stimulates BCL2/p53 binding, which may demonstrate an effective and innovative strategy for the treatment of hematologic malignancies [34]. 

## 5. Conclusions

The results of the study on genes revealed that they are critical and involved in disease progression. In addition, the relative expression fold indicates the cell’s response to the treatment, as the selected genes are essential for cell proliferation and cell apoptosis. Our findings might be used as a guideline for selecting the appropriate treatment that fits patients in clinics. Also, the hematological parameters such as lymphocytes, neutrophils, and platelet count seemed to be abnormal in some CML patients who received particular treatments. These results recommend having some precautions after using cancer therapy, as it might impact the immune system or coagulation. 

## Figures and Tables

**Table 1:** Shows the sequence of nucleotides for the designed primers used in this study:

Gene	Forward	Reverse
B-actin	CGG GAC CTG ACT GAC TAC C	TGA AGG TAG TTT CGT GGA TGC
Bcl2	ATC GCC CTG TGG ATG ACT GAG T	GCC AGG AGA AAT CAA ACA GAG GC
Cip2a	TGC GGC ACT TGG AGG TAA TTT	AGC TCT ACA AGG CAA CTC AAG C
Pp2a	GGT GGT CTC TCG CCA TCT ATA G	CTG GAT CTG ACC ACA GCA AGT C
Wt1	GAA AAT AGG GGA TGG TCC AG	CAA TGG ATT TCC TCA CCC AG

**Table 2:** Correlation between the studied genes under different treatments for CML patients.

Gene Expression	Treatments		
	HU	IM	Combination
PP2A vs. CIP2A	−0.18433	−0.30453	−0.20241
BCL2 vs. CIP2A	−0.33206	0.991093	−0.40355
BCL2 vs. PP2A	−0.22445	−0.35362	−0.64193
WT1 vs. PP2A	0.324249	−0.54996	−0.76978
WT1 vs. CIP2A	−0.6094	0.939511	0.509226
WT1 vs. BCL2	−0.34197	0.967696	0.579199

## Data Availability

The datasets generated during and/or analyzed during the current study are available at request from the corresponding author.

## Abbreviations

2G TKI	Second-generation tyrosine kinase inhibitors
AP	Accelerated phase
Ara-C	Cytosine arabinoside
ATP	Adenosine triphosphate
B-ALL	B acute Lymphoblastic Leukemia
Baso	Basophils
BCL-2	B-cell lymphoma 2
BCR	B-cell receptor
BM	Bone marrow
BP	Blastic phase
cDNA	Complementary deoxyribonucleic acid
CGL	Chronic granulocytic leukemia
CIP2A	Cancerous inhibitor of protein phosphatase 2A
CML	Chronic myeloid leukemia
C-MYC	Cellular-Myelocytomatosis
CP	Chronic phase
DNA	Deoxyribonucleic acid
E2F1	E2 promoter binding factor 1
EDTA	Ethylenediamine tetra acetic acid
Eosino	Eosinophils
ERK1/2	Extracellular signal-regulated kinases 1 and 2
FDA	Food and Drug Administration
FISH	Fluorescence in Situ Hybridization
GRB2	Growth Factor Receptor Bound Protein 2
HB	Hemoglobin
Hb F	Fetal hemoglobin
HFLC	Lymphocytes with strong fluorescence
HHT	Hereditary hemorrhagic telangiectasia
HU	Hydroxyurea
IM	Imatinib
JAK2	Janus kinase 2
Lymph	Lymphocyte
MCH	Mean corpuscular hemoglobin
MCHC	Mean corpuscular hemoglobin Concentration
MCV	Mean cell volume
Mono	Monocytes
MPV	Mean platelet volume
MRD	Minimal residual disease
mRNA	Messenger ribonucleic acid
NCB	Normal cord blood
Neutro	Neutrophils
NRBC	Nucleated red blood cell
PADs’	PP2A-activating drugs
PCR	Polymerase chain reaction
PCT	Plateletcrit
PCV	Packed Cell Volume
PDGFR	Platelet-derived growth factor
PDGFRA	Platelet-derived growth factor receptor A
PDW	Platelet distribution width
PLT	Platelet
PP1	Protein phosphatase 1
PP2	Protein phosphatase 2A
RDW-CV	Red blood cell distribution width -cell volume
RDW-SD	Red blood cell distribution width—standard division
RNA	Ribonucleic acid
RNA Seq	RNA sequencing
RNR	Ribonucleotide reductase
RR M2 subunit	Ribonucleotide reductase regulatory subunit M2
RT-qPCR	Quantitative reverse transcription—Polymerase chain reaction
RT-PCR	Reverse transcription- Polymerase chain reaction
SSC	Side scatter light
STAT	Signal transducer and activator of transcription 5
TKI	Tyrosine kinase inhibitors
Tm	Melting temperatures
UT	Untreated
WBC	White blood cell
WHO	World health organization
WNR channels	White cell nucleated
WDF channels	WBC differential
WT1	Wilms’ tumor protein
